# In–situ strain control in epitaxial silicon carbide compound semiconductor

**DOI:** 10.1038/s41598-024-80810-7

**Published:** 2024-12-05

**Authors:** Behzad Jazizadeh, Maksym Myronov

**Affiliations:** Department of Physics, The University of Warwick, Coventry, CV4 7AL UK

**Keywords:** 3C–SiC, MEMS, CVD, Strain, Heteroepitaxy, Materials science, Materials for devices

## Abstract

Residual strain in an epilayer grown on a foreign wafer induces epiwafer’s bow, that is often considered undesirable. Wafer bow however, can be advantageous because both the direction and magnitude of strain are vital for the fabrication of various Micro Electro Mechanical Systems (MEMS), such as resonators. Here strain control is reported for highly mismatched heteroepitaxy of cubic silicon carbide (3C–SiC) compound semiconductor on silicon (Si), a prized functional material, dependent solely on carbon to silicon ratio (C/Si) during growth. While Si–rich condition enhances growth and generates positive curvature i.e. tensile strain, C–rich condition suppresses growth and produces negative curvature i.e. compressive strain. An optimum region emerges with virtually no strain and superior crystallinity. Our findings are significant for the knowledge of heteroepitaxy of 3C–SiC and may be broadened to heteroepitaxy of other compound semiconductors.

Thin film cubic silicon carbide (3C–SiC) compound semiconductor is the only polytype of SiC that can be grown on a Si substrate. Technologically this implies fast, low cost and industrially scalable growth^1–8^; but also means the generation of residual strain due to lattice mismatch^9^ and difference in the coefficients of thermal expansion^10^. As strain refers to the elastic distortion of crystal lattice under intrinsic stress gradient, strain-relaxation within the epilayer leads to unwanted defects and unintentional curvatures. While tremendous amount of research has been dedicated to the understanding and reduction of defects in 3C–SiC thin films on Si substrates over the years^7–22^ not much attention has been given to curvature (strain–stress) in the thin films. It is essential to note that while the presence of defects is harmful for applications in electronics, it is the curvature (bow) resulting from residual strain that is most devastating for epiwafers processing during microfabrication of electronics and other devices, but could be beneficial for Micro Electro Mechanical Systems (MEMS) applications.

Curvature in epiwafers (wafer bow), depending on severity, may lead to thickness nonuniformity in successively deposited layers, degradation in lithography accuracy, difficulty in handling the wafer by semiconductor manufacturing equipment with robotised wafer handling, and delamination followed by cracking of photoresist or deposited layers^23,24^. Little work has been channelled towards managing or minimizing residual strain. Most important are the attempts to address the issue focusing on deposition parameters such as the choice of precursors, variation in temperature and controlling the deposition pressure^25–27^. However, these efforts have resulted in degradation of crystalline quality, and there are serious doubts about their repeatability and possible effects on other material properties such as elemental composition. Most recently, there had been reignited strives to tackle the issue via introducing high levels of (aluminium) dopants into the epilayers^28,29^, changing substrate orientation (from 001 to 111)^30–32^, modifying film thickness^32^ or post–growth enhancements via microstructures^33^. These attempts though, are either short of complementary and independent investigation of the material system or require the substrate or the epilayer to be altered by additional growth steps or fabrication processing. Any solution to the problem at hand needs to be suitable for large–scale epitaxial wafer manufacturing as well as fast inexpensive processing. This article, for the first time, presents the strain adjustment of 3C–SiC thin films simply by way of tuning the carbon (*C*) content; here the *C* content refers to the *C/Si* atomic ratio between C–containing and Si–containing precursors in a gas phase during (one–step) growth^34^ by chemical vapour deposition (see Methods, for more details). In-situ variation of *C* content has led to the transformation of strain in undoped 3C–SiC epilayers from tensile to zero to compressive, effectively taking in all possible states of residual strain.

An implication of this study is that: to overcome high levels of residual strain in heteroepitaxial layers it is not necessary to grow thicker films that in turn require longer growth times and larger amounts of precursors. In fact, thinner epilayer not only drives down the cost of growth, but also simplifies micromachining of MEMS and NEMS structures. This is even more advantageous for a material such as silicon carbide that possesses mechanical and electrical properties that are superior to their counterparts, and for which thicker epilayer (longer growth) can thus be avoided. This however means that measurable wafer bow would be a remote possibility, as was the case in this study. As a result, the fabrication of microcantilevers provided the solution to measurability problem.

Presented in Fig. 1a an actual 3C–SiC/Si (001) epiwafer and the sample’s schematic as it is cleaved along the 〈110〉 plane (parallel to primary flat of 100 mm diameter Si(001) wafer), respectively. All microcantilevers were patterned in order to be released with their lengths along the cleaving direction. Both curvature (left y–axis) and the deflection (right y–axis) profiles of the microcantilevers (Fig. 1b) are shown, as functions of *C* content, normalized to the thickness of epilayers: *k*_*norm*_ (referred to as *k*) and *z*_*norm*_ (referred to as *z*), respectively. Data points in the deflection plot refer to the maximum deflection, at the tip of the cantilever.

Datasets in this and other sections of the article were normalized to sample thickness by subtracting the mean thickness value from each individual thickness data. This difference was then divided by the standard deviation, as given below:

1 $$\:X:=\frac{X-m}{s}$$

where $\:X$ are data values, $\:m$ is the mean of the data and $\:s$ is calculated as:

2 $$\:s=\sqrt{E\left[{X}^{2}\right]-{\left(E\left[X\right]\right)}^{2}}$$

where *E[X*^*2*^*]* is the mean of the squared data and *(E[X])*^*2*^ is the square of the mean of data ($\:m=E\left[X\right]$). This process generated normalized (dimensionless) values corresponding to each thickness value that were utilized as coefficients for further calculations where normalization deemed to be needed.

Curvature and deflection reach the highest positive values at lesser *C* content and drop to the lowest negative values at greater *C* content exhibiting large tensile and compressive strains, respectively. This is also supported by the scanning electron microscopy (SEM) of the arrays of microcantilever with different lengths, for positive and negative curvature, presented by insets I and III of Fig. 1b, respectively. A transition region of minimal strain exists, with C/Si values mid–way between tensile and compressive in Fig. 1b. A closer look at this region reveals that, even in a region of virtually strain–free epilayers, there exists some residual strain that is of a compressive nature. A further look at Fig. 1b inset II shows that while almost all cantilevers look straight, the longest one undergoes slight compression which suggests the presence of a critical aspect ratio for strain–free beams. Note that sign determination refers to the deflections away from substrate as positive or + z, and towards the substrate as negative or -z; as schematically visualized next to SEM images.

**Fig. 1:**
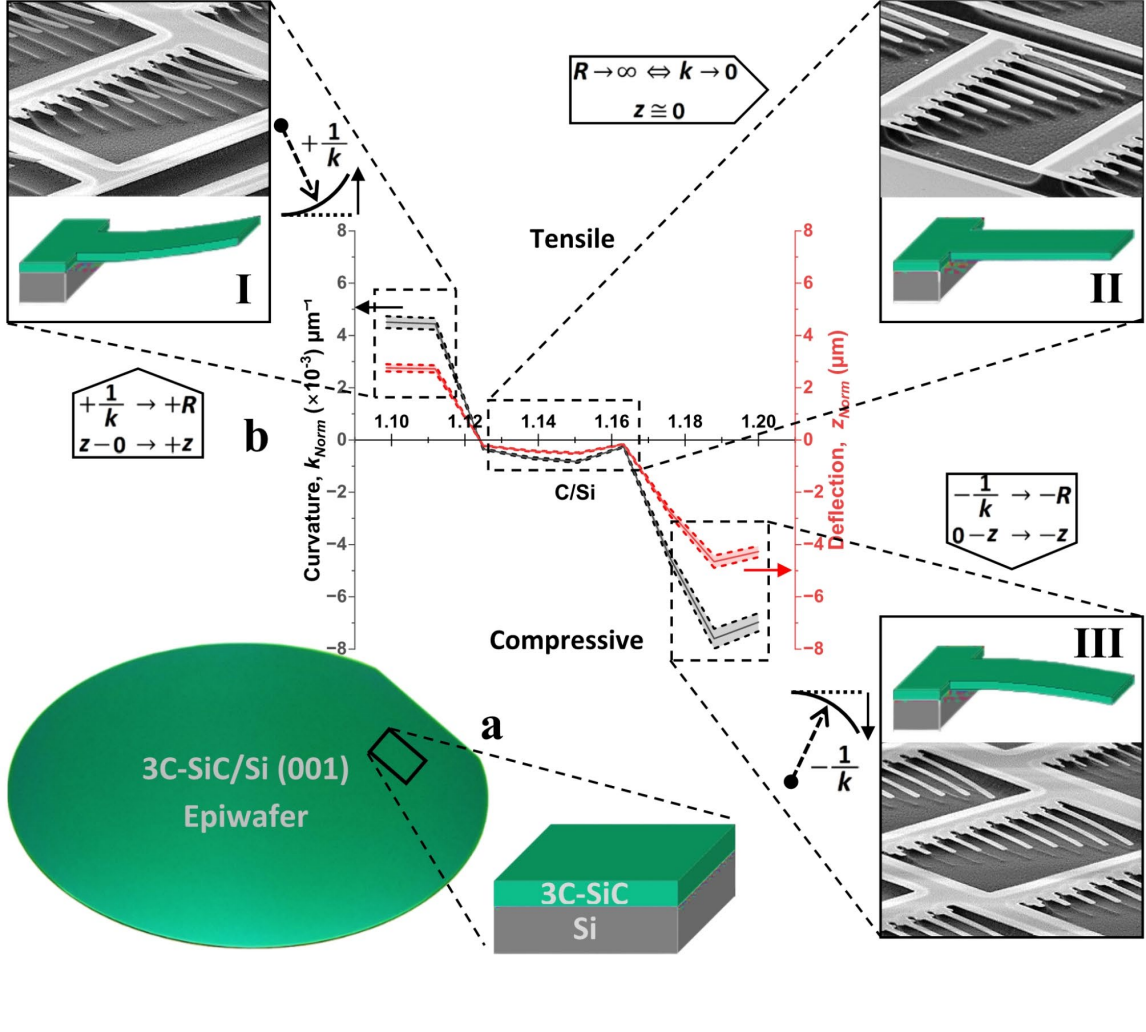
Schematics of 3C–SiC/Si (001) epiwafer, sample and cantilevers, and elastic measurements. (**a**) Optical photograph of an actual 3C–SiC/Si (001) epiwafer, one of the series with different *C* contents grown in house for this study, from which a standard sample is cleaved parallel to the large wafer flat. (**b**) A superposition plot of *k* (left Y–axis) and $\:z$ (right Y–axis) as a function of *C* content (shown as *C/Si*). **I**,** II** and **III** Insets show cantilever schematics along with SEM images of cantilever arrays with state of curvature in tensile (thickness 329 nm), neutral (thickness 302 nm) and compressive (thickness 273 nm), respectively. Dashed lines indicate the 5% error margin.

Reduction in thickness of the epilayers as a function of increasing *C* content is demonstrated in Fig. 2a. In Fig. 2b the left (vertical) axis represents full width at half maximum (*FWHM*) values that were obtained from rocking curve (RC) x–ray diffractometry (XRD) measurements of (002) plane and normalized to the thickness of the epilayers; while the right axis shows *R* values normalized to the thickness. Additional *C* content beyond *C/Si* ≈ 1.165 affects both crystalline and mechanical properties corresponding to increase in both *FWHM* and *R* values. Insets I and II of Fig. 2b clearly visualize the (002) RC curve peak shift to the left and right from the relaxed position, for the tensile and compressive *R*, respectively, matching the Si–rich and C–rich growth regimes. Presented in Fig. 2c, for comparative purposes, is a characteristic high-resolution HR–XRD coupled scan of the Si–rich, C–rich, and optimum region, as previously specified in Fig. 2a, b. The substrate Bragg peak, Si (004), is kept for reference and others from multiple crystal planes, where the Bragg peaks would have been positioned, are marked for clarity.

**Fig. 2:**
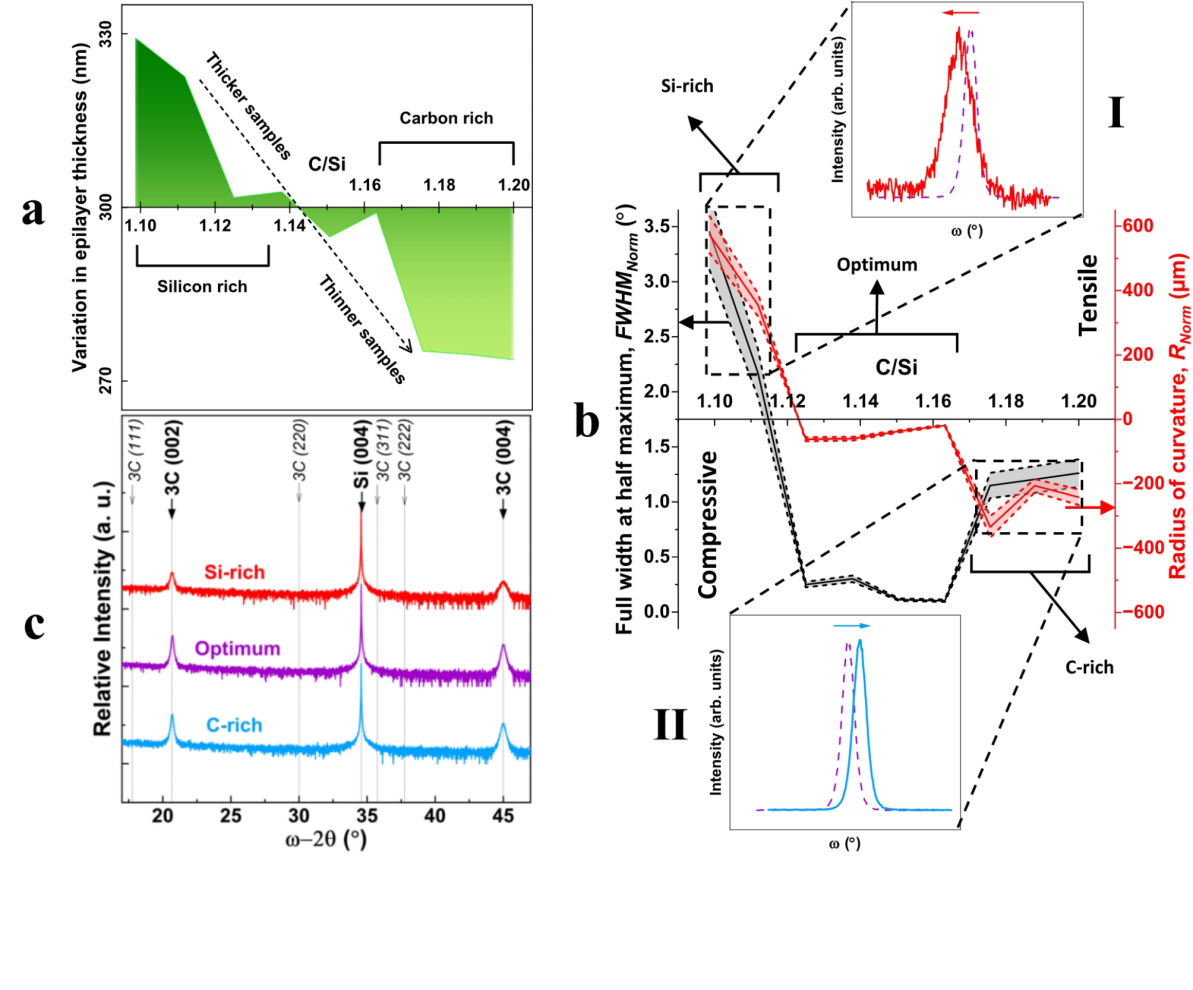
Thickness, curvature variation and crystal quality. (**a**) Thickness measurements as a function of *C* content (shown as *C/Si*) denoting thicker at the Si–rich and thinner at the C–rich end. (**b**) A superposition plot of (002) RC FWHM (left Y–axis) and R (right Y–axis), as a function of *C* content (shown as *C/Si*). **I**, Inset showing a typical RC curve at the Si–rich end, shifted to lower angles indicating uniform tension, and **II**, Inset showing a typical RC curve at the C–rich end, shifted to higher angles indicating uniform compression. Both down–shift and up–shift of RC angles are referenced to the relaxed RC curve shown as dashed lines in insets **I** and **II**. (**c**) Plot of a representative XRD spectra of the 3C–SiC/Si (001), for the Si–rich, optimum region and C–rich offset at the top, middle and bottom of the plot, respectively. Available Bragg peaks for 3C (002), Si (004) and 3C (004) are marked by bold large fonts with thick arrows, while Bragg peaks that are unavailable (noted above the XRD spectra) are marked by italic small fonts with thin arrows.

Note the optimum range that exists following the switch in signs of both curvature and deflection from tensile to compressive and then the sharp drop into more compressive values. Depicted by inset II of Fig. 1b this optimum region (or optimum range) with *C* contents within the interval of 1.125 ≤ *C/Si* ≤ 1.165 was observed, according to the SEM imaging, to have very little to no deflection, and is identified as an interval with the curvature and deflection values being an order of magnitude smaller compared to the rest of the profile. This showcases a region of virtually no-bending for 3C–SiC epilayers. The deflection and curvature profiles embolden similarities with the variation in epilayers’ thickness, seen in Fig. 2a.

As all epiwafers have similar growth time, the drop in thickness of 3C–SiC means reduced growth rate, as a result of increased *C* content. When the precursors decompose near the growth surface (Si substrate), methyl (CH_3_) groups are introduced into surface reactions; they are relatively stable transient intermediates and remain mobile across the growth surface. For the duration of their stay on the growth surface, CH_3_ groups effectively block Si atoms from bonding to C atoms, see Fig. 3a. These CH_3_ groups form methane (CH_4_) or ethane (C_2_H_6_) upon recombination with H atoms or neighbouring CH_3_ groups, respectively (see Fig. 3b); and eventually leave the surface (see Fig. 3c). It is speculated that the excess C atoms (blocked from bonding with Si atoms) will be incorporated into the lattice structure, as apparent from Fig. 2a.

**Fig. 3:**
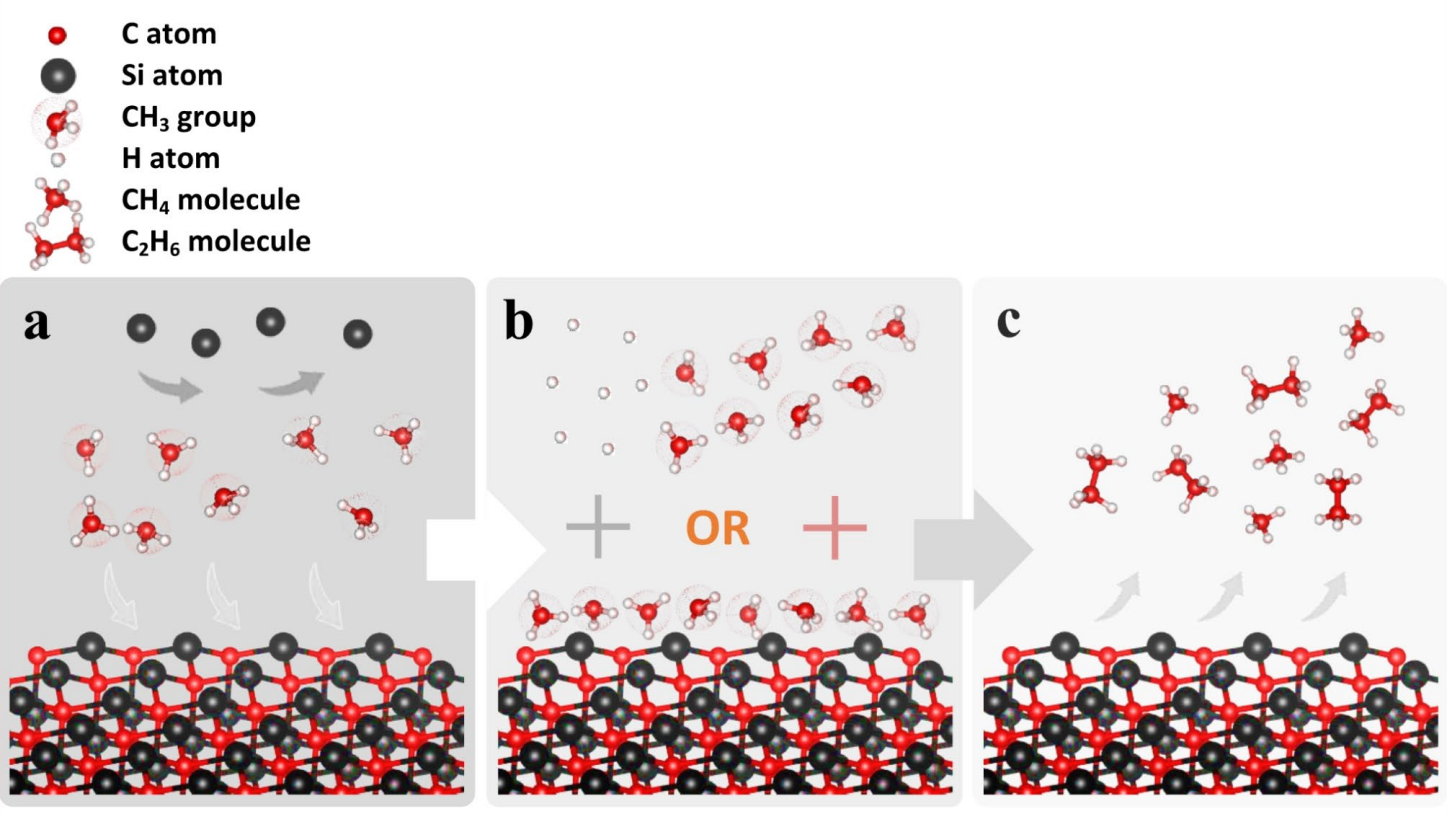
The role of CH_3_ groups in the C–﻿rich growth region. (**a**) stable CH_3_ groups arrive at the surface and block Si atoms. (**b**) CH_3_ groups are then joined by either H atoms or other CH_3_ groups nearby. (**c**) CH_3_ groups, following near–surface reactions, leave either in the form of CH_4_ or C_2_H_6_ molecules.

The curvature–deflection behaviour of the epilayers, and in particular the transition from tensile to compressive strain, is comparable with the overall trend in *FWHM* values (Fig. 2b left vertical axis). The largest *FWHM* values (of Si–rich region) correlate with the largest tension (the most positive values), while the rise of the *FWHM* values within the C–rich agree with the largest compression (the most negative values). In the same way, the *FWHM* values of the optimum region (implying the highest crystal quality of all samples) are in fact positioned where the minimally bent 3C–SiC epilayers show only slight compression that are (on average) an order of magnitude lower than both the positive and negative peaks. All this is while only the 3C–SiC (002) and (004) Bragg peaks were observable, across all samples; and the absence of Bragg peaks associated with planes other than those equivalent to 3C–SiC crystal structure implies that the 3C–SiC epilayers is monocrystalline, regardless of the evolving *FWHM* values as a function of *C* content.

**Fig. 4:**
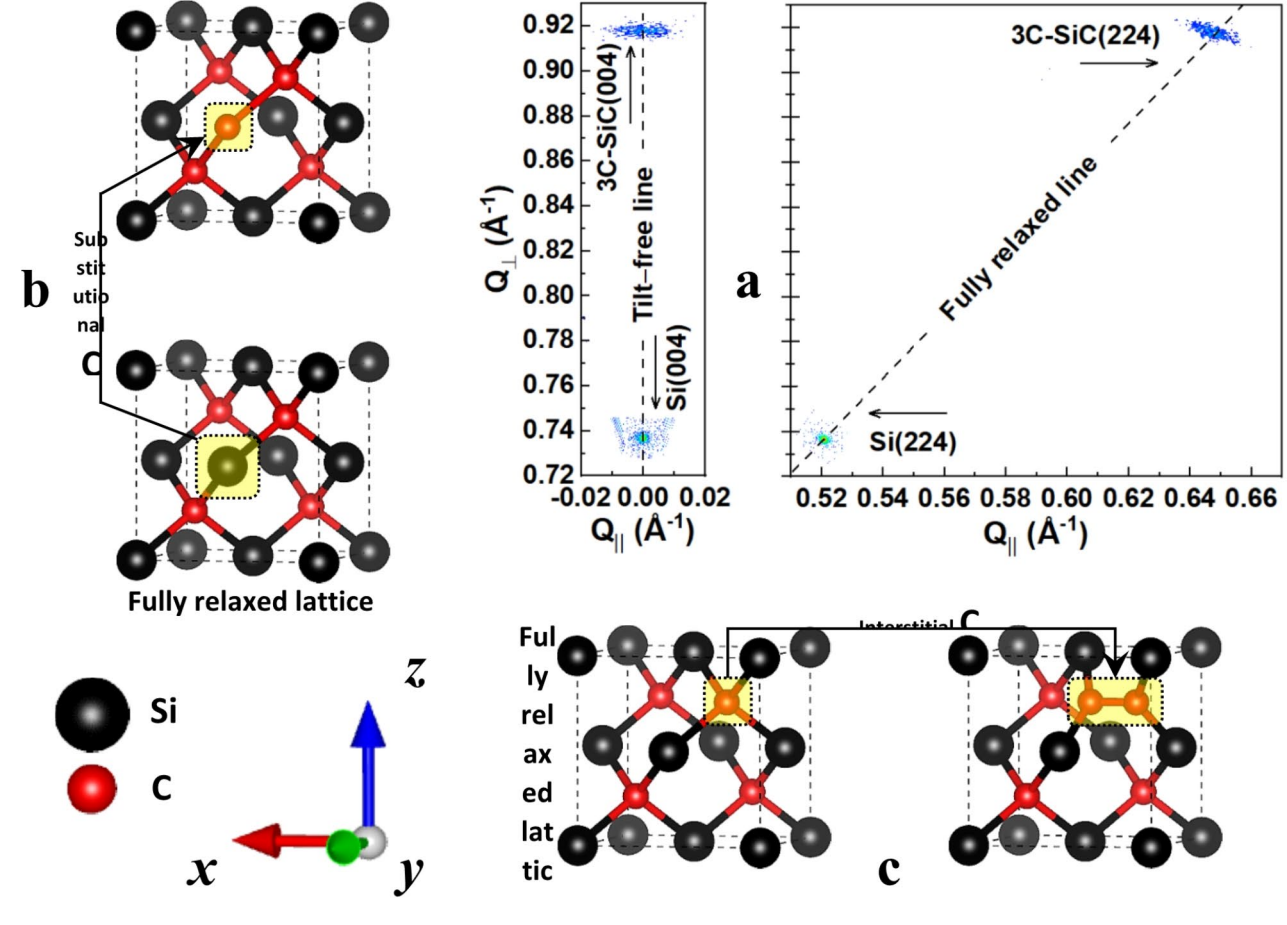
Reciprocal space map, substitutional and interstitial defects. (**a**) Reciprocal space map (RSM) of a typical strained epitaxial 3C–SiC (here at *C/Si* ≈ 1.2); with the (004) and (224) maps showing the tilt and strain, respectively. Dashed lines represent the reference positions for the tilt–free and strain–free (fully relaxed) epilayers, respectively. (**b**) Schematic showing the possibility of substitutional C incorporation, as *C/Si* ratio increases. (**c**) Schematic showing the possibility of interstitial C incorporation, as *C/Si* ratio increases.

Here we described the state of curvature–deflection of 3C–SiC/Si epilayers, representing the residual strain, as controlled only using C/Si (or rather the C content) during growth. Reciprocal space mapping (RSM) of the (004) and (224) planes, as shown in Fig. 4a, are a confirmation that while the epilayers with non-optimum C/Si ratio (see Fig. 4a, right hand side for C/Si ≈ 1.2) are strained, they are free of tilt (see Fig. 4a, left hand side symmetrical scan). The intensity distribution of the asymmetrical 3C-SiC (224) scan visualizes the compressive shift, away from the fully relaxed line (delineated with the diagonal dashed line); which is in agreement with the curvature and deflection measurements (Fig. 2b, inset II) and the SEM images of the cantilever profiles (Fig. 1b, inset III). The evolution of the state of strain in 3C-SiC/Si(001) as a function of C content is hypothesized to be the result of structural defects that are generated within the epilayers during growth. These defects whose impact is enough to deeply transform mechanical properties, but not sufficient to fundamentally change the monocrystalline characteristics of the epilayers, seem to alter the three regions Si–rich, optimum and C–rich differently. As C content increases it is postulated that these structural defects, in the C–rich, are attributed to the C content that is integrated into the crystal lattice, the nature of which is still under investigation. Of all the possible types of crystallographic defects due to excess C, two main arrangements seemed to the authors as the most probable ones. Those are schematically presented in Fig. 4b and c, in the forms of substitutional C and interstitial C, respectively; suggesting the possibilities of having either shallow or deep electronic states within the bandgap. Further studies are underway to obtain a deeper understanding of the exact types and natures of the defects. To the best of our knowledge, this is the first time that a material has been developed with strain properties tuneable in such a way that all three states of tensile, zero, and compressive are accessible. Results and discussions in this study can be further extended to the heteroepitaxial growth of other technologically important compound semiconductors, both current and emerging, such as the likes of gallium nitride (GaN) and aluminium nitride (AlN), as well as boron arsenide (BAs) and boron nitride (BN), respectively.

## Methods

### Growth of epilayers

High quality 3C–SiC epilayers, variable in thickness, have been grown at low temperatures using a single-step epitaxy in an industry standard Si based reduced pressure chemical vapor deposition (RP–CVD) system. Growth was carried out on on-axis 100 mm diameter 525 μm thick Si(001) wafers within an ASM Epsilon 2000 cold wall system, capable of growth on wafers of up to 200 mm diameter. Growth rates of over 10 μm/hr was obtained. The Si wafers were loaded into the CVD growth chamber at 900 °C. The temperature was then rapidly raised to ~ 1000 °C to thermally desorb the native oxide from Si surface. The 3C–SiC growth immediately followed at below 500 Torr chamber pressure, and with no carbonization steps involved. More details about the growth can be found in reference^34^. A mixture of Trimethylsilane (TMS) and Dichlorosilane (DCS) was used as the growth precursors. To realize optimal growth conditions and control the 1:1 stoichiometry of 3C–SiC, the following crucial formulation was calibrated and maintained:

3 $$\:\frac{C}{Si}=\frac{3\times\:TMS}{DCS+TMS}$$

From which the variation of *C/Si* was achieved by carefully adjusting the TMS flow ratio.

### Fabrication of cantilevers

**Fig. 5:**
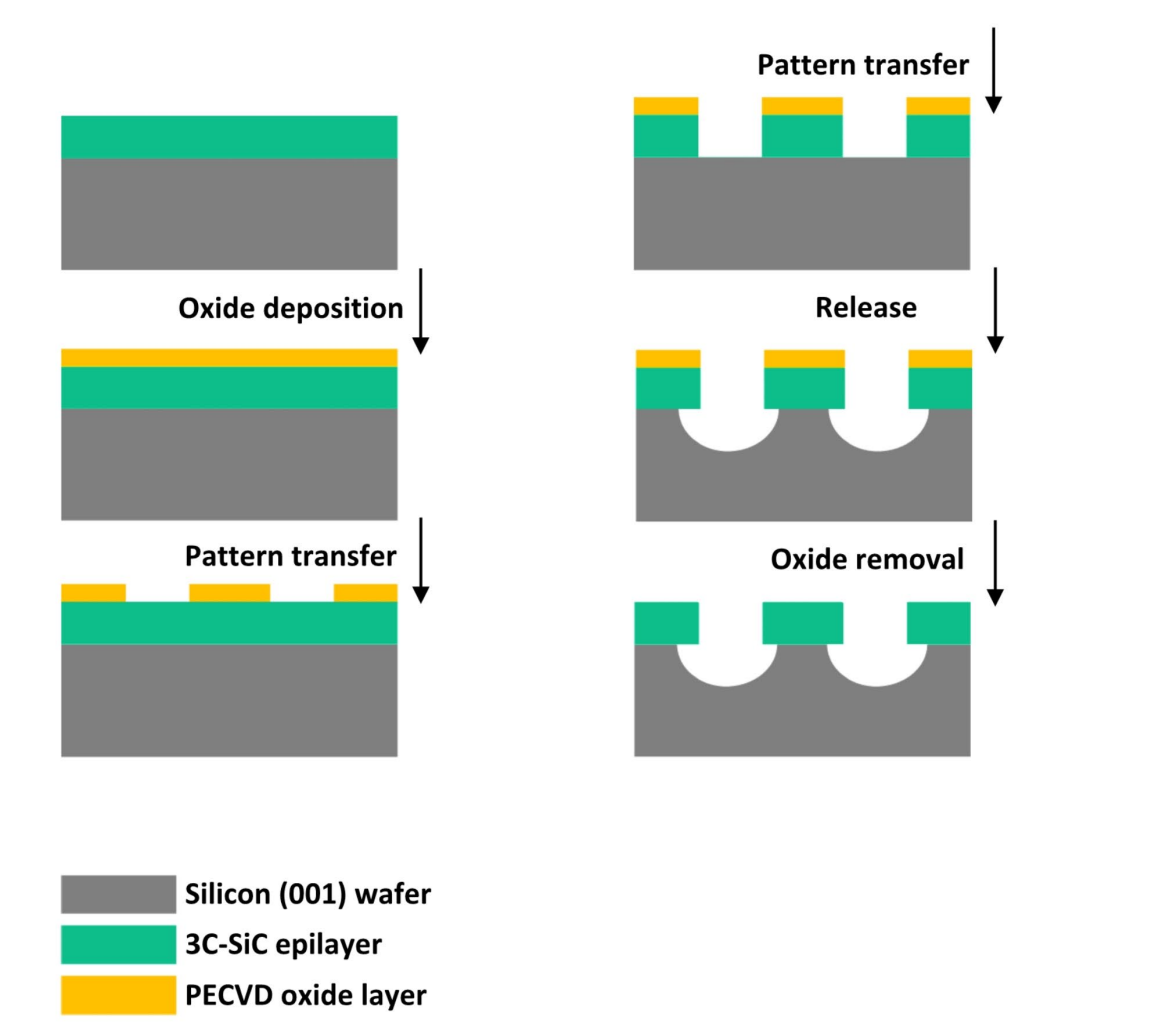
Cantilever fabrication process. All–dry steps low temperature fabrication process for microcantilevers leading to stiction–free highly etch-selective suspended structures.

Arrays of 3C–SiC test devices (microcantilevers) with variable aspect ratios were suspended from Si substrate, and examined in terms of their mechanical parameters. The fabrication process for all samples was performed in parallel and at the same time, with identical steps and parameters; this eliminated any possible differences that could appear from one sample to another, among other factors, as a result of difference in the duration of exposure to outside contaminants. As shown in Fig. 5 the fabrication process started with a low temperature silicon dioxide (SiO_2_) layer of similar thickness to the carbide (3C–SiC) layer, deposited to be used as etch mask. Samples were then patterned with the layout of the microdevices using direct write lithography exposing a thin layer of photoresist (resist), followed by resist development. Device patterns were then transferred onto the oxide layer inside the reactive ion etcher using a 200 *W* RF power Trifluoromethane / Argon (CHF_3_ / Ar) plasma, with 17.7 / 20.1 standard cubic centimeter per minute (*sccm*) flow respectively. The final pattern transfer and release of the devices, took place inside a inductively coupled plasma (ICP) deep reactive ion etcher. Etch parameters were initially set to 2000 *W* (source RF), 100 *W* (electrode RF) to generate a Sulfur hexafluoride / Oxygen (SF_6_ / O_2_) plasma, with 55 / 10 *sccm* for 3C–SiC, and then finally set to 2000 *W* (source RF) with electrode RF source turned down to zero to strike a SF_6_–only plasma for Si; thereby developing an undercut to suspend the devices. Resonators were designed as cantilever beams (single clamped); with one end of the beam fixed (anchored) and one end free to move. The microcantilevers were patterned in 1D arrays with fixed width (5 *µm*) and variable lengths (7 *µm* to 98 *µm*).

### Characterization

Structural properties of the 3C–SiC crystals were analysed using X–ray diffractometer, including X–ray diffraction (XRD), rocking curves’ analysis (RC) and reciprocal space mapping (RSM) analysis all on PANalytical X’Pert Pro MRD. Images were obtained from the Zeiss SUPRA 55VP FEGSEM scanning electron microscope (SEM). Thickness of epilayers was measured by spectroscopic ellipsometry (SE), on Accurion Nanofilm EP4, and confirmed by Fourier transform infrared (FTIR) on Bruker Vertex 70v, and X–ray reflectivity (XRR). Deflections of the microcantilevers were measured using white light interferometry.

Tip deflections, as the maximum cantilever deflection, were utilized in the deflection plot. The data points for curvature values were taken from a triplet set of deflection measurements, separately spaced along the length of the cantilever. Cantilevers with similar lengths and from the same position were chosen from each array. Five arrays of cantilevers were randomly chosen for five measurements; and the final deflection value was taken as the average of those measurements. Curvature (k) was then approximated using the Menger definition and method^35,36^, as:

4 $$\:k=\frac{1}{R}$$

And R is the radius of the circle that passes through the triplet of data points, and is obtained using:

5 $$R=\frac{abc}{4\,S}$$

where *a*, *b* and *c* are respectively the side lengths corresponding to vertices *A*, *B* and *C* (the data points); and *S* is the surface area of the triangle created by the three sides *a*, *b* and *c*. A more detailed formulation is given in reference^35^. Measurement error from one sample cantilever to the next fell within one standard deviation.

## Data Availability

All data are available in the main text and supplementary materials. Any additional data are availablefrom the corresponding author upon request.
